# The Indian Hemp

**Published:** 1843-05

**Authors:** 


*The Indian Hemp.*—Feb. 22.—Mr. Ley this evening communicated a paper on the *cannabis Indica,* from which it appears that, although the hemp is rejected from our official preparations in England, it is, and has been for a long series of years, in constant use as a popular remedy throughout the East. It exhales a powerful narcotic odour, and the branches are gelatinous to the touch, with a resinous secretion, which is collected when the seed is found (as the plant is then in the greatest perfection), and is sold under the name of *churrus*, the shoots from which the resin has *not* been collected, are cut, dried, and sold as gunjah. Although hemp is no longer used medicinally in England, there is an old Act of Henry the Eighth yet in force, by which it is forbidden to be soaked in ponds or running streams where cattle drink. The older writers speak of it as a violent poison, and state that the water in which it has been soaked produces its effects almost as soon as drank. The resin of the cannabis Indica is in general use as an intoxicating agent from the furthermost confines of India to Algiers. If this resin be swallowed, almost invariably the inebriation is of the most cheerful kind, causing the person to sing and dance, to eat food with great relish, and to seek aphrodisiac enjoyment. The intoxication lasts about three hours, when sleep supervenes; it is not followed by nausea or sickness, nor by any symptoms, except slight giddiness, worth recording. These effects are much modified in this country, and much less marked, possibly from the length of the voyage rendering the article deteriorated in value. The subsequent effects are depression of spirits and relaxation of the muscles in a marked degree; the free perspiration on the skin, and the increase of appetite, have made some old rheumatic persons speak of it as the elasticity of youth.

Mr. Ley draws a comparison between the effects produced by opium and those caused by the cannabis Indica, the result of which induces him to give the preference to the latter, its influence being excited more kindly and more gratefully on the system. It has proved of service in cholera and rheumatism, but it is in spasmodic and convulsive diseases that it is most eminently useful. In tetanus it has been the means of cure in the majority of cases, both in men and horses, and it has much relieved the horrors of hydrophobia, although not averted the fatal termination. It is useful, also, in chorea, spasmodic asthma, and delirium tremens, and generally wherever opium is indicated. Mr. Ley considers from this that it will prove a direct antidote, the first of its class, to strychnia, one of the most violent poisons nature affords.

*Medico-Botanical Society.—Lond. Lancet.*

